# A methylomics‐associated nomogram predicts recurrence‐free survival of thyroid papillary carcinoma

**DOI:** 10.1002/cam4.3388

**Published:** 2020-08-11

**Authors:** Hengyu Chen, Xianxiong Ma, Ming Yang, Mengyi Wang, Lei Li, Tao Huang

**Affiliations:** ^1^ Department of Pancreatic Surgery Union Hospital Tongji Medical College Huazhong University of Science and Technology Wuhan China; ^2^ NHC Key Laboratory of Hormones and Development Tianjin Institute of Endocrinology Tianjin Medical University Chu Hsien‐I Memorial Hospital Tianjin China; ^3^ Department of Gastrointestinal Surgery Union Hospital Tongji Medical College Huazhong University of Science and Technology Wuhan China; ^4^ Department of Breast and Thyroid Surgery Union Hospital Tongji Medical College Huazhong University of Science and Technology Wuhan China

**Keywords:** DNA methylation, nomogram, RFS, signature, TPC

## Abstract

**Background:**

Thyroid papillary carcinoma (TPC) is the most common type of thyroid cancer (TC). The prognosis of TPC patients with tumor‐cell metastasis is poor. Therefore, this study aims to develop a model for predicting TPC patients' recurrence‐free survival (RFS).

**Methods:**

We included 546 TPC patients who were clinically and pathologically diagnosed with TPC. The methylation biomarkers that associate with RFS were explored. These 546 samples were divided into training dataset (first 70%) and validation dataset (remaining 30%) randomly. The training dataset was used to identify prognostic biomarkers and construct risk prediction model, in addition, the validation dataset was used to verify the predictive performance of the model. We used Cox proportional hazard analysis and the least absolute shrinkage and selection operator (LASSO) Cox regression analysis to identify the significant predictive biomarkers, and establish the relapse risk prediction model from the identified biomarkers.

**Results:**

A 6‐DNA methylation signature yielded a high evaluative performance for RFS. The Kaplan‐Meier analysis indicated that the 6‐DNA methylation signature could significantly distinguish the high‐ and low‐risk patients in training, validation and entire sets. In addition, a nomogram was constructed based on risk score, metastasis status and residual tumor status, and C‐index, receiver operating characteristic (ROC) and the calibration plots analysis which demonstrated the good performance and clinical utility of the nomogram.

**Conclusions:**

The results suggested that the 6‐DNA methylation signature is the independent prognostic marker for RFS and functioned as a significant tool for guiding the clinical treatment of TPC patients.

## Graphical abstract

We constructed a nomogram that combined both the 6‐DNA methylation signature and the conventional clinicopathological factors to predict 1‐, 3‐, and 5‐year recurrence‐free survival (RFS). Which may be an independent prognostic marker for RFS and functioned as a significant tool for guiding the clinical treatment of thyroid papillary carcinoma patients.

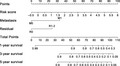

## 1 INTRODUCTION

Thyroid cancer (TC) is the most common endocrine carcinoma with a rapid increase in incidence worldwide.^1^, ^2^ Thyroid papillary carcinoma (TPC) is the most common type of thyroid cancer and accounts for more than 75% of TC^3^ and has the best overall prognosis.^4^ Before tumor‐cell metastasis, 5‐year survival rate of TPC patients was over 95% after combined therapy including thyroidectomy, radioactive iodine (RAI), and thyroid‐stimulating hormone (TSH) suppression therapy. However, metastasis of TPC led to high recurrence.^5^ Various classification systems on the basis of crucial patient‐specific and tumor‐specific characteristics have been used to predict the prognosis of patients with TC and assess disease‐free survival and cause‐specific mortality in patients with TC. These classification systems have been applied to guide aggressive treatment for high‐risk patients and to avoid excessive treatment for low‐risk patients.^6^, ^7^ However, there are few studies investigated whether the numerous molecular markers found in TC have prognostic values.

It was reported that genes controlled by DNA methylation are associated with tumor progression.^8^ An increasing number of reports have suggested that DNA methylation may function as potential prognostic biomarkers. For instance, methylation alteration of SHANK1 may serve as a predictive, diagnostic and prognostic biomarker for chronic lymphocytic leukemia.^9^ Methylation of PCDH19 may predict poor prognosis of hepatocellular carcinoma.^10^ However, the defects of these previous studies included small sample size and unstable predictive robustness. Besides, only a few genes were researched and the studies lacked combined and systematic genome‐wide methylation analysis. Therefore, the whole‐genome methylation profiles of tumor samples from patients with TPC through The Cancer Genome Atlas (TGGA) databases were analyzed and a risk prediction model for recurrence‐free survival (RFS) based on methylation of DNAs was constructed and tested in our study.

## 2 MATERIALS AND METHODS

### 2.1 DNA methylation data of TPC tissues

We downloaded all TPC DNA methylation data measured by illumina Human Methylation 450 BeadChip (illumina Inc) and clinical data by R TCGAbiolinks package.^11^ DNA methylation levels were expressed as *β* values, and calculated as *M*/(*M* + *U* + 100). *M* represents the signal from methylated beads, and *U* refers to the signal from unmethylated beads at the targeted CpG site. We only included DNA methylation data from patients whose survival information was available. The relevance between DNA methylation levels and the RFS was explored. In total, 546 samples with 485,577 DNA methylation sites were included in our study. These 546 samples were classified into training dataset (first 70%) and validation dataset (remaining 30%) randomly. The training dataset was employed to identify and construct a prognostic signature, and the validation dataset were exploited to verify the predictive performance of the signature. LASSO method was used to screen the key methylation sites that have predictive value for RFS. LASSO Cox regression model was conducted through a publicly available R package‘glmnet’^12^ for 1000 iterations.

### 2.2 Data processing, normalization, and identification of differentially expressed methylation sites

The data were preprocessed before building the prediction model. Methylation sites whose beta value is not available (NA) in greater than 10% of the total samples were excluded from the study. For the missing beta value more than 10% of the total samples, we assumed the NA data by “impute.knn” function from Impute package.^13^ Finally, the data normalization was conducted based on “betaqn” function from wateRmelon package.^14^

In addition, all the samples were separated into early TPC group and advanced TPC group. The standardized beta was transformed to *M* value based on the formulation: *M* = log(*β*/(1 − *β*)). *M* value was calculated to standardize the difference caused by different probes. Then, *M* value was performed to identify differentially expressed methylation sites between early and advanced group based on “dmpFinder” function from minfi package.^15^

### 2.3 Statistical analyses

The univariate Cox proportional hazard analysis was implemented to screen methylation sites that are significantly (*P* < .05) associated with TPC patient's RFS. Subsequently, the LASSO Cox regression analysis was implemented to further identify the candidate methylation sites influencing the RFS of TPC patients. Thereafter, the candidate methylation sites were employed to construct risk prediction model. Finally, AUC was also applied to assess the model performance. The model with a better predictive performance was screened base on AIC (Akaike information criterion) value, the smaller the AIC, the better performance the model is. A formula was created to determine RFS risk scores for every patient on the basis of this model. Patients with TPC were separated into high‐ and low‐risk group via the median score as the cutoff. Kaplan‐Meier survival analysis was exploited to calculate the prediction value of the model for the risk of TPC patients' RFS. Kaplan‐Meier curves were drawn through the “survival” package.^16^ Finally, the receiver operating characteristic (ROC) analysis was implemented by the “survival ROC” package with a categorical variable for application in predicting TPC patients' RFS.

### 2.4 Gene set variation analysis (GSVA)

To detect the DNA methylation biomarker‐relevant signaling pathways, single sample gene sets enrichment analysis (ssGSEA) was executed in the light of TCGA TPC mRNA dataset through GSVA package.^17^ Patients with TPC were divideded into high‐ and low‐risk group via the median score as the cutoff. A *P* value of <.05 was considered significant.

### 2.5 Construction of the nomogram

A nomogram was performed based on the “rms” R package. Factors that were used to construct the final multivariate Cox proportional hazard model were applied to develop nomogram. C‐index, ROC as well as calibration plots were performed to evaluate the prognostic accuracy of the nomogram. The result of the nomogram was showed in the calibrate curve, and the 45° line implied the best prediction.

## 3 RESULTS

### 3.1 Clinical characteristics of the study populations

**Figure 1:**
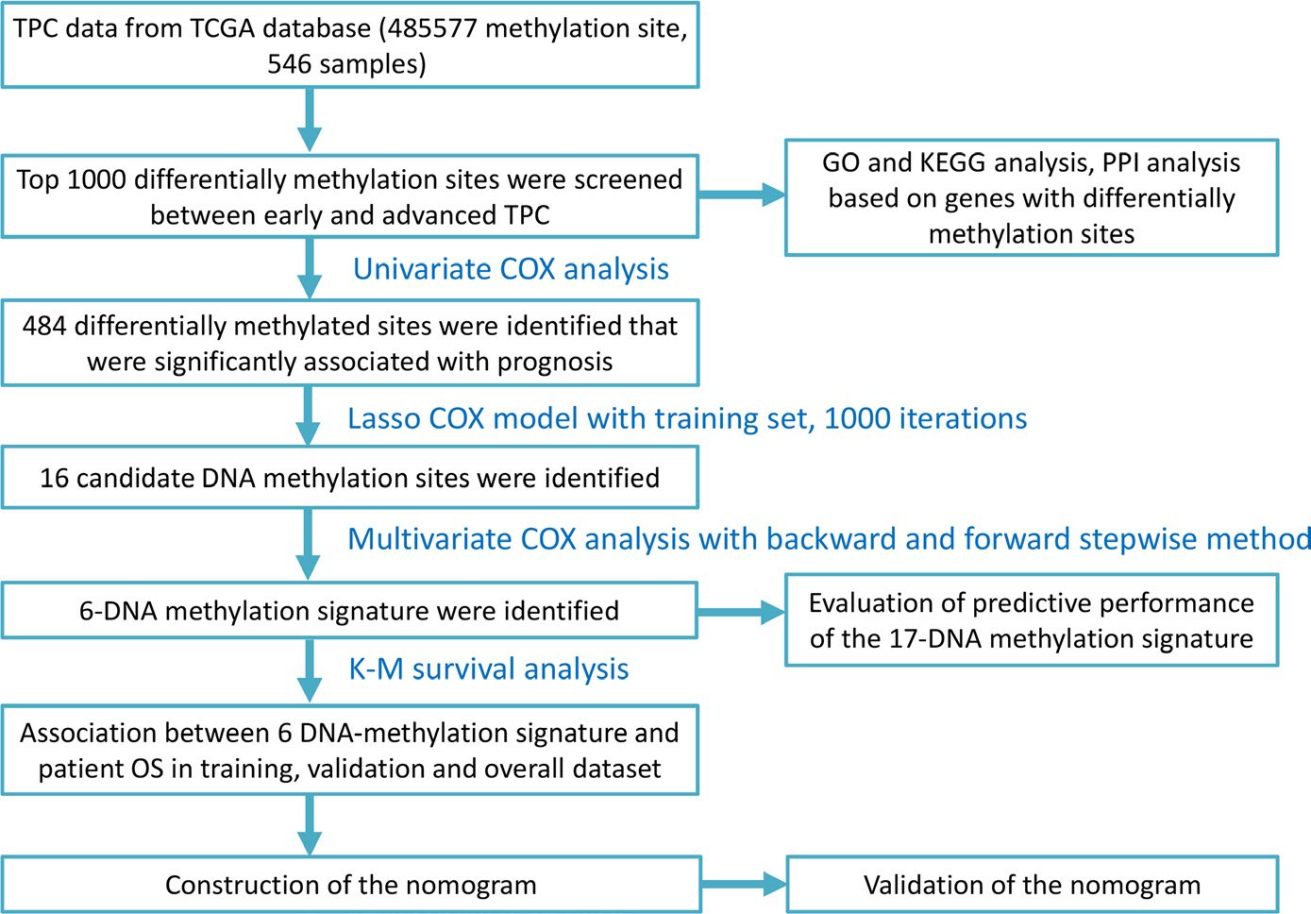
Flow chart of the this study

We included 546 TPC patients who were clinically and pathologically diagnosed with TPC, with 145 (26.56%) males and 401 (73.44%) females. The median age at diagnosis was 46 years (range, 15‐89) and the median RFS were 911 days. The 3‐year RFS rate of all patients was 37.36%. The pathologic stage was defined based on the American Joint Committee on Cancer (AJCC) Cancer staging manual. The stage of TPC patients ranged from I to IV, and 317 (58.06%) patients in state I, 57 (10.44%) patients in stage Il, 119 (21.79%) patients in stage Ill, and 53 (9.7%) in stage IV. Histological _type of the study patients included TPC‐ Classical/usual 401 (73.44%), TPC‐ Follicular (≥99% follicular patterned) 106 (19.41%), and TPC‐ Tall Cell (≥50% tall cell features) 39 (7.14%), respectively. Patients were divided into four groups based on residual tumor, that is R0 420 (76.92%), R1 52 (9.52%), R2 3 (0.55%), and other 71 (13.0%). In addition, medical disorder history of TPC patients included normal group, lymphocytic thyroiditis group, nodular hyperplasia group, other group. Normal group was the most common type 306 (56.1%) (Table 1). Figure 1 displays the workflow chart of the present study, which described the experimental procedure of the present study.

**Table 1:** Clinical characteristics of included patients with thyroid papillary carcinoma (TPC)

Characteristics	Total	Training dataset (n = 383)	Testing dataset (n = 163)
Sex			
Female	401 (73.44)	277 (72.32)	124 (76.07)
Male	145 (26.56)	106 (27.68)	39 (23.93)
Histological type			
Thyroid papillary carcinoma classical/usual	401 (73.44)	276 (72.06)	125 (76.69)
Thyroid papillary carcinoma follicular (≥99% follicular patterned)	106 (19.41)	76 (19.84)	30 (18.4)
Thyroid papillary carcinoma tall cell (≥50% tall cell features)	39 (7.14)	31 (8.09)	8 (4.91)
Stage			
Stage I	317 (58.06)	216 (56.4)	101 (61.96)
Stage II	57 (10.44)	38 (9.92)	19 (11.66)
Stage III	119 (21.79)	86 (22.45)	33 (20.25)
Stage IV	53 (9.7)	43 (11.2)	10 (6.1)
Residual tumor			
R0	420 (76.92)	286 (74.67)	134 (82.21)
R1	52 (9.52)	39 (10.18)	13 (7.98)
R2	3 (0.55)	2 (0.52)	1 (0.61)
Other	71 (13.0)	56 (14.6)	15 (9.2)
Age			
<50	312 (57.14)	221 (57.7)	91 (55.83)
≥50	234 (42.86)	162 (42.3)	72 (44.17)
Ethnicity			
Hispanic or latino	38 (6.96)	28 (7.31)	10 (6.13)
Not hispanic of latino	396 (72.53)	285 (74.41)	111 (68.1)
Other	112 (20.5)	70 (18.3)	42 (25.8)
Medical disorder history			
Normal	306 (56.1)	214 (55.9)	92 (56.4)
Lymphocytic thyroiditis	75 (13.7)	51 (13.3)	24 (16.7)
Nodular hyperplasia	71 (13)	53 (13.8)	18 (11.04)
Other	94 (17.2)	65 (17.0)	29 (17.8)

### 3.2 Identification of 6 methylation sites signature

**Figure 2:**
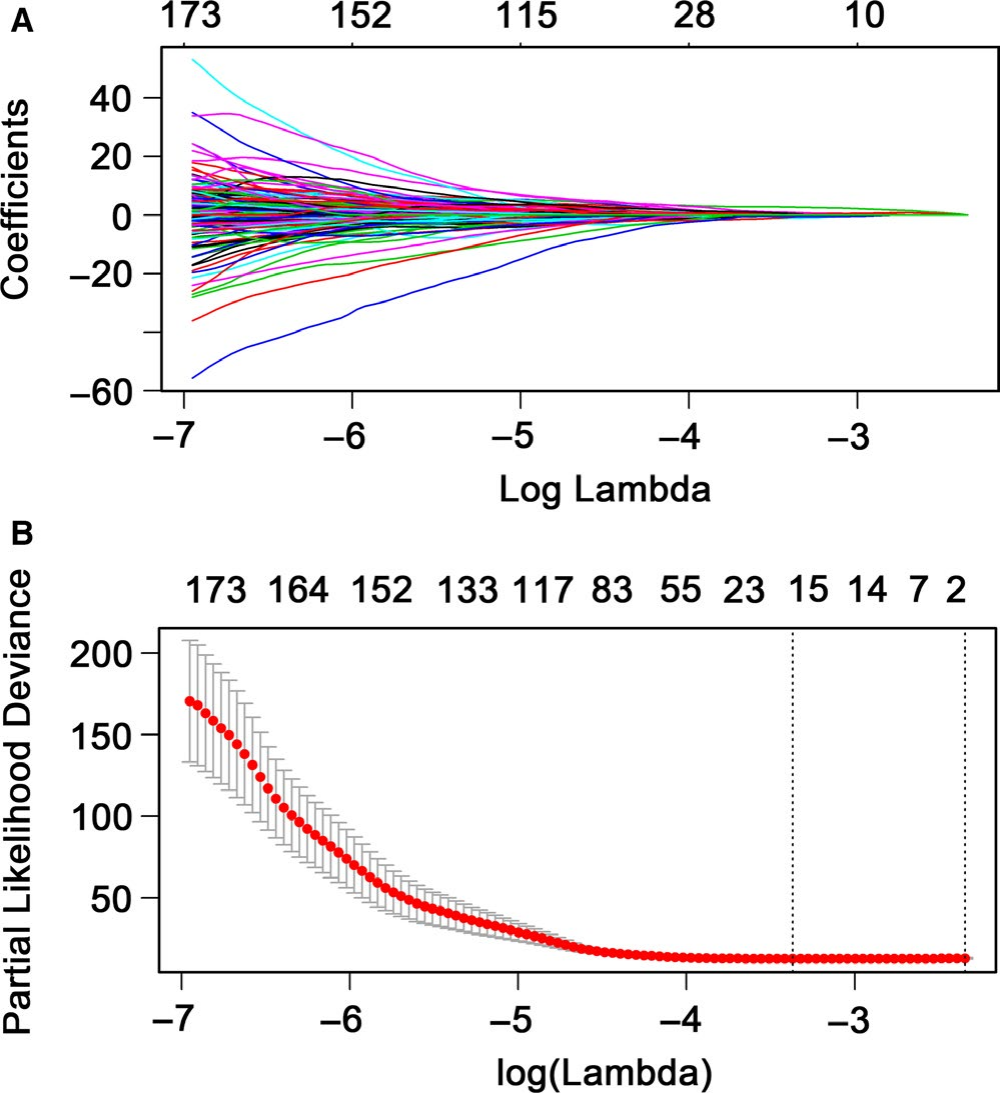
Candidate methylation sites selection using the least absolute shrinkage and selection operator (LASSO) Cox regression model. A, 10‐fold cross‐validation for tuning parameter selection in the LASSO model through minimum criteria (the 1‐SE criteria). B, LASSO coefficient profiles of the 448 methylation sites. A coefficient profileplot was created against log(lambda) sequence. Vertical line was plotted at the value selected based on 10‐fold cross‐validation and optimal lambda yielded 16 non‐zero coefficients

**Figure 3:**
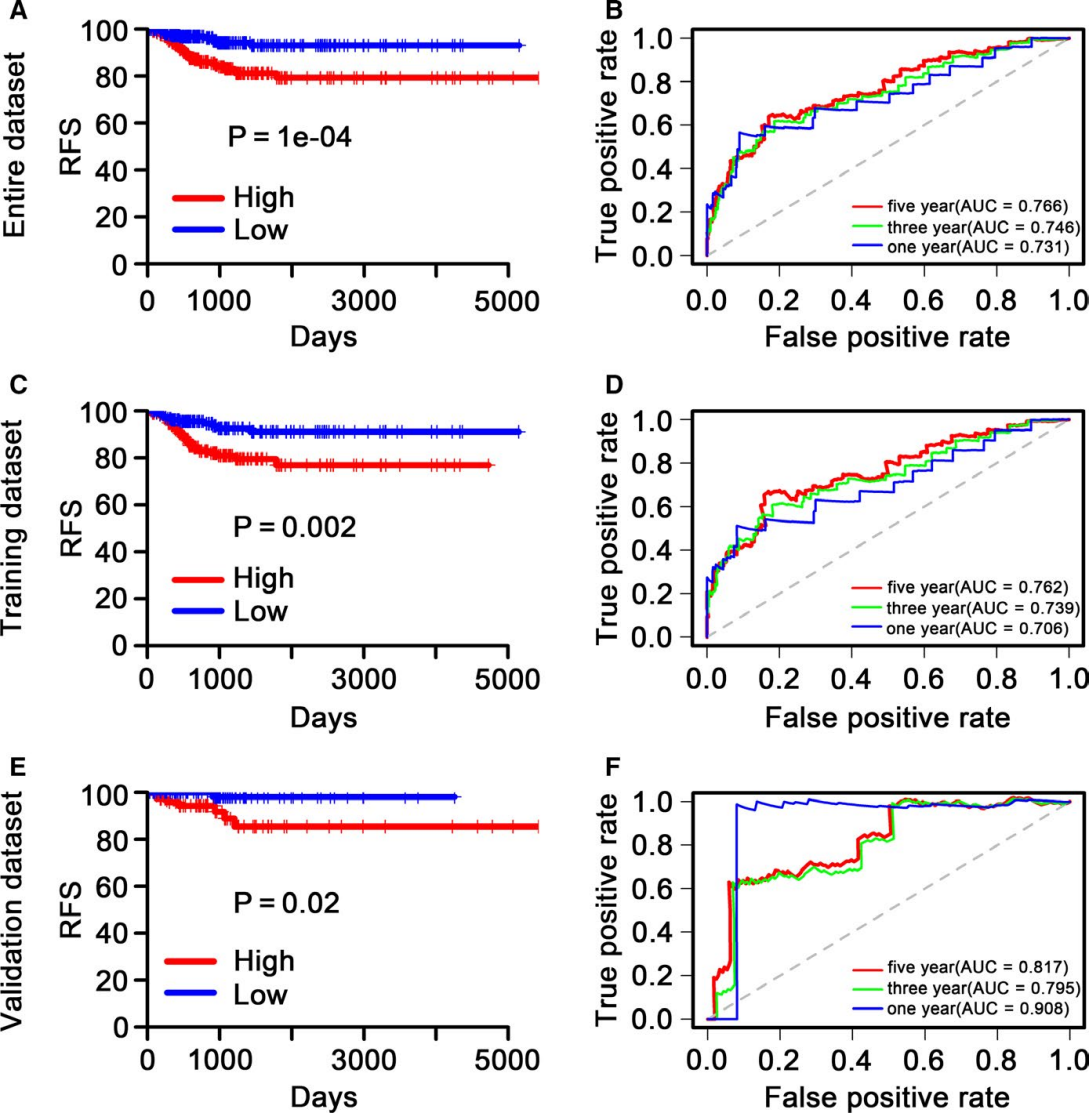
Boxplots of methylation β values against risk group in the entire dataset. Patients with thyroid papillary carcinoma (TPC) were divided into high‐ and low‐risk group based on the median score as the cutoff. “High” and “Low” referred to the high‐risk and low‐risk cohorts, respectively. The differences between the two cohorts were assessed by Mann‐Whitney *U* test, and *P* values were showed below the graphs

Further analysis based univariate Cox proportional hazard regression model showed a total of 448 DNA methylation sites that were significantly associated with RFS of patients (*P* < .05) (Table S1). Subsequently, LASSO Cox regression model was developed and identified 16 methylation sites as the candidate prognostic factors for RFS (Figure 2A,B). Finally, a risk score formula based on 6 methylation sites that were identified by multivariate Cox proportional hazard regression analysis was constructed: Risk score = −5.367*cg17749033 + 1.619*cg24221648 + 2.334*cg01664864 + 1.873*cg09578568 − 3.486*cg24051057 + 5.693*cg05972352. Obviously, the hypermethylation levels of cg24221648, cg01664864, cg09578568, and cg05972352 were associated with a higher risk of recurrence or mortality caused by disease progression. Nevertheless, the hypomethylation levels of cg17749033 and cg24051057 were related to a higher risk of recurrence or mortality caused by disease progression (Figure 3; Figures S1 and S2).

**Figure 4:**
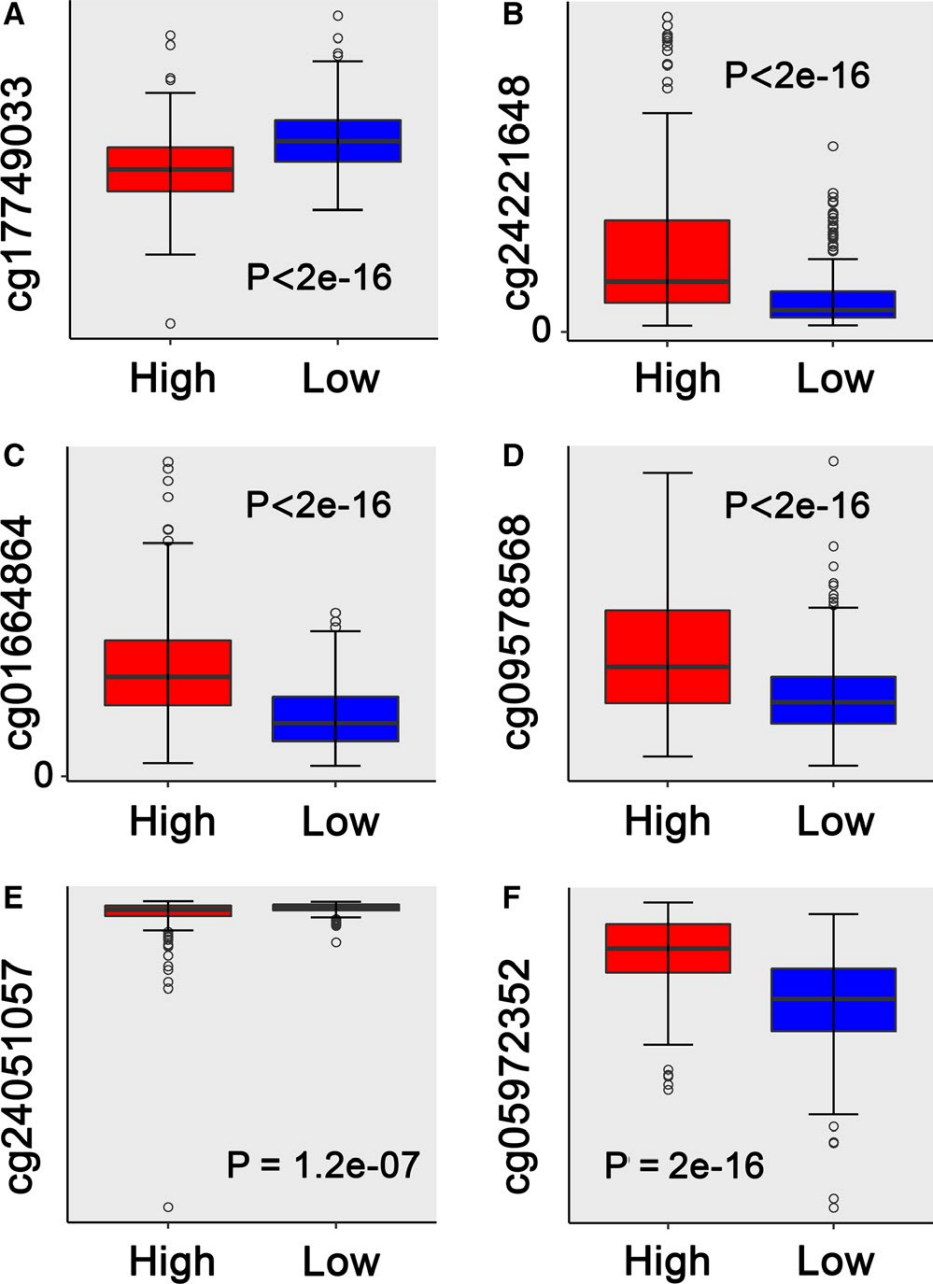
Kaplan‐Meier and receiver operating characteristic (ROC) analysis of patients with thyroid papillary carcinoma (TPC) in training, testing, and entire dataset, respectively. A, C and E, Kaplan‐Meier survival curve in training, testing and entire dataset, respectively. Kaplan‐Meier analysis with two‐sided log‐rank test was carried out to assess the differences in recurrence‐free survival (RFS) between the low‐risk and high‐risk TPC patients. Patients with TPC were separated into high‐ and low‐risk group via the median score as the cutoff. B, D and F, ROC curves in training, testing and entire dataset, respectively. 1‐, 3‐, and 5‐year ROC curves of the 6‐DNA methylation signature were employed to assess the performance in predicting TPC patients' RFS. The higher the AUC value, the better the performance of the ROC curve

Kaplan‐Meier analysis was exploited in the training and validation datasets as well as the entire dataset to measure the RFS of patients in the low‐ versus high‐risk group which were separated based on 6‐DNA methylation signature. The patients with high‐risk scores group had poorer RFS in entire dataset (*P* = .0001) (Figure 4A), a similar result was displayed in the training dataset (*P* = .002) (Figure 4C), and validation dataset (*P* = .02) (Figure 4E).

### 3.3 Evaluation of the predictive value of the 6 methylation sites signature using ROC analysis

We described the predictive value of the 6‐DNA methylation signature in predicting RFS using a time‐dependent ROC curve. The higher the AUC value, the better the prediction of the 6‐DNA methylation signature. The AUC of the 6‐DNA methylation signature at 1, 3, and 5 years in the entire dataset were 0.731, 0.746, and 0.766, respectively (Figure 4B). A high predictive performance was also yielded in training dataset (0.706, 0.739, 0.762) (Figure 4D) and validation dataset (0.908, 0.795, 0.817) (Figure 4F), which indicated that the 6‐DNA methylation signature had potential to serve as a hallmark for predicting TPC patients' RFS in clinical applications.

**Figure 5:**
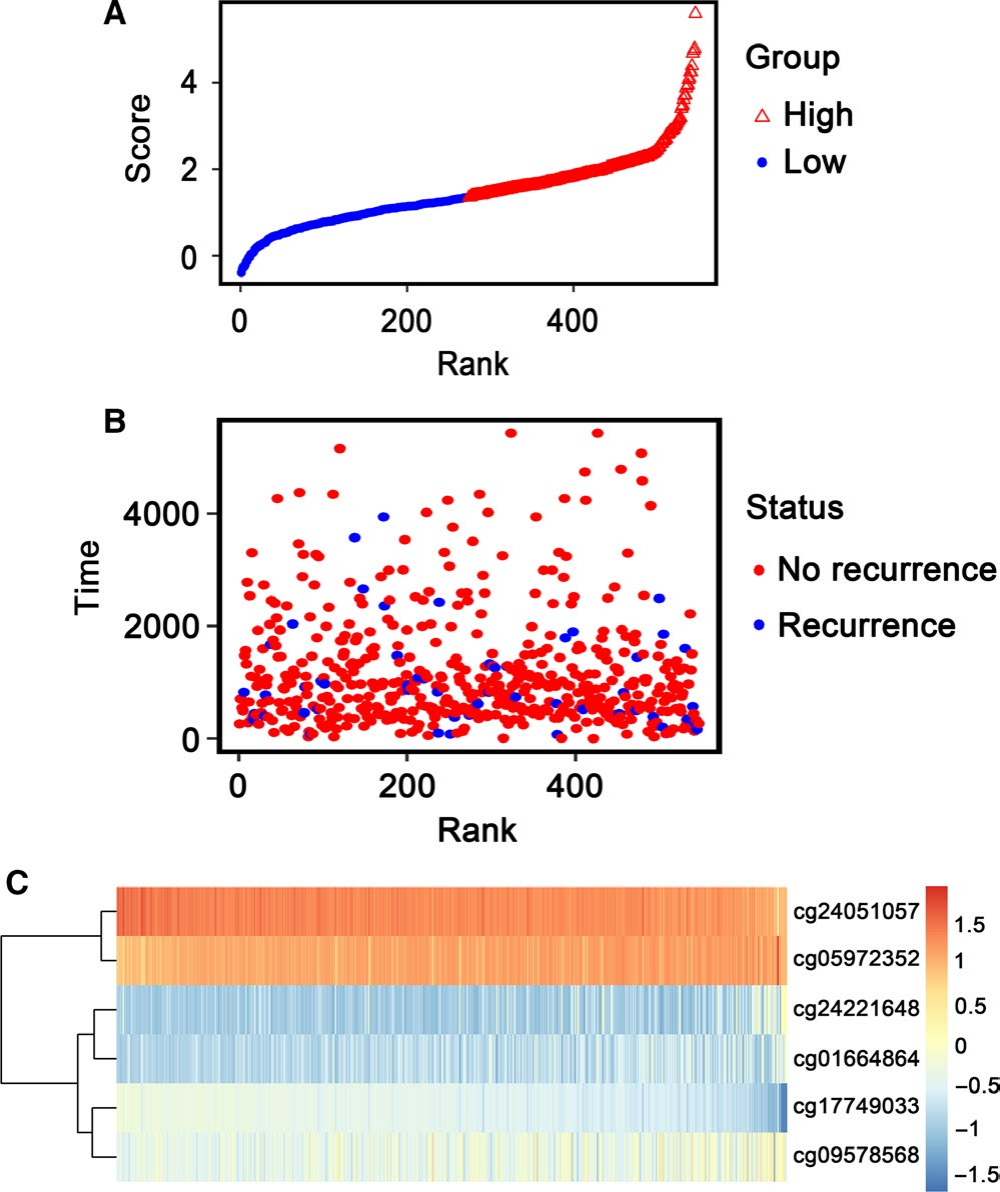
Methylation risk score analysis of 546 thyroid papillary carcinoma (TPC) patients in the entire dataset. A, methylation risk score distribution based on the rank of risk score. Median risk score was used as the cut‐off point. The triangle represented the high‐risk group, the circle represented the low‐risk group. B, Survival status of TPC patients. The red circles represented recurrences, the green circles represented no recurrences. C, Heatmap of 6 methylation sites expression profiles of TPC patients. Each row of the heat map represented a radiomics feature and each column represented a patient. The Score difference of each radiomics feature between high risk and low risk group can be seen from the heat map

In addition, patients were ranked according to their risk scores (Figure 5A), and a dotplot was drew according to their survival status (Figure 5B). Result indicated that the low‐risk group had a lower mortality rate than the high‐risk group. Heatmap of 6 methylation sites sorted by risk score was presented in Figure 5C, which was consistent with our previous boxplot.

Besides, subgroup analysis was performed by various clinicopathological factors including age, gender, stage, histologic type, residual tumor, ethnicity, and medical history, which also yielded a good predictive performance in most subgroups (Figures [Link], [Link], [Link], [Link], [Link], [Link], [Link]), indicating that the 6‐DNA methylation signature displayed good performance for predicting TPC prognosis in the majority of the sub‐groups.

### 3.4 Exploration of the 6‐DNA methylation signature‐related biological pathways

**Figure 6:**
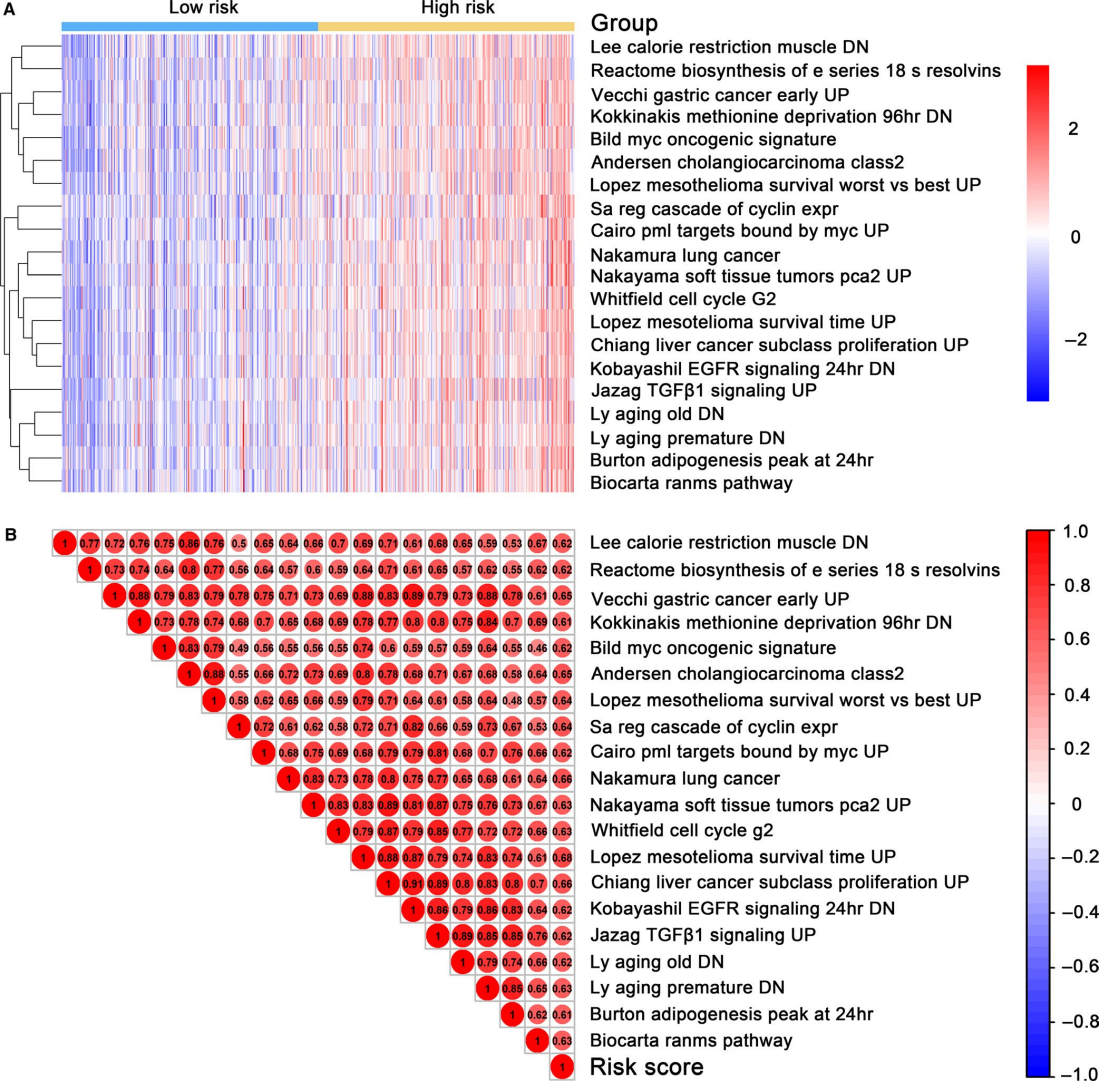
Identification of the 6‐DNA methylation signature‐related biological pathways. A, Heatmap of top 20 enriched pathways related to high‐risk group. B, Association graph between risk scores and top 20 pathways. Rows stood for pathways, and columns stood for patients. Each grid stood for ascore of pathway activity calculated by single‐sample GSEA. No further adjustment of the single sample gene sets enrichment analysis (ssGSEA) score was performed

TPC patients were divideded into high‐ and low‐risk group using the median score as the cutoff. Top 20 DNA methylation risk score related pathways that were more activated in the high‐risk groups than that in low‐risk groups are summarized in Figure 6A (Table S2). In addition, the same trend between the enriched pathways and risk score was further verified in Figure 6B.

### 3.5 Nomogram development and assessment

**Figure 7:**
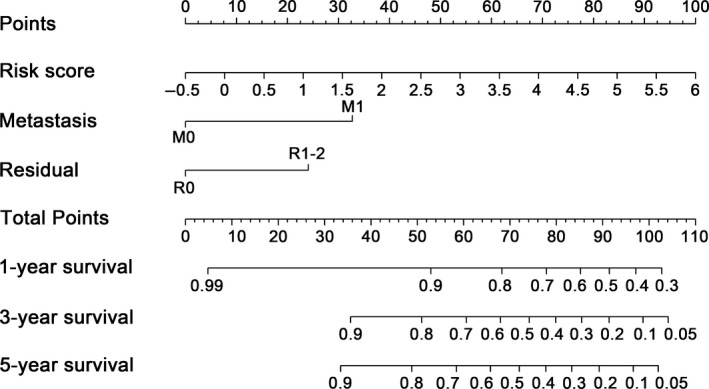
Methylation nomogram for the prediction of thyroid papillary carcinoma (TPC) patients' RFS. The nomogram was built in the entire group based on the methylation risk score, metastasis status, and residual status. C‐index, receiver operating characteristic (ROC) and the calibration plots were used as evaluation indicators for the nomogram

**Figure 8:**
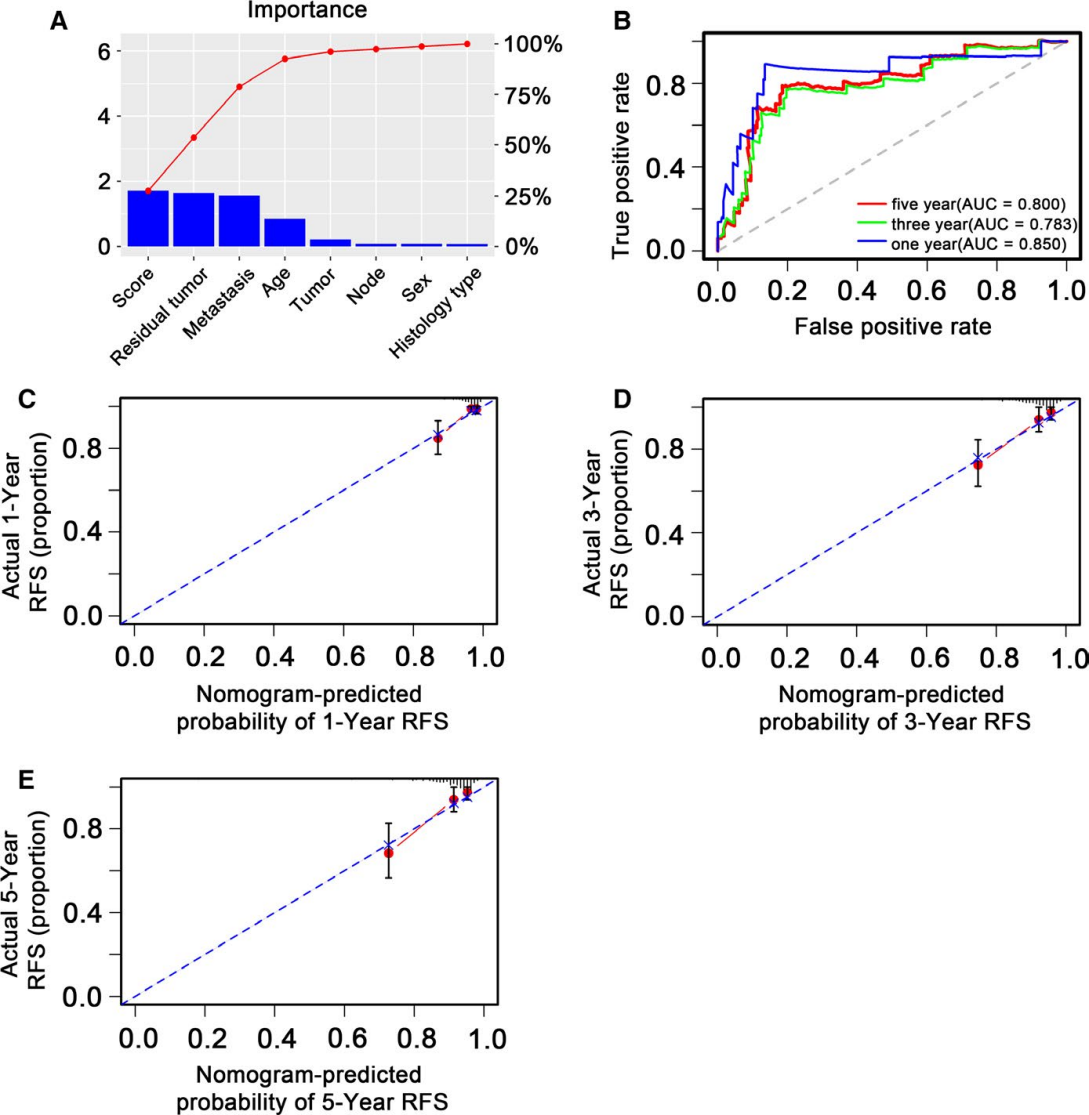
Confirmation of methylation nomogram in entire dataset. A, Barplot of importance of each clinical factor. B, 1‐, 3‐, and 5‐year receiver operating characteristic (ROC) curves for the methylation nomogram. The horizontal axis represented clinical factors, the vertical axis represented the percentage of importance. C, D and E, stand for the 1‐, 3‐, and 5‐year nomogram calibration curves, respectively. The closer the dotted line fit is to the ideal line, the better the predictive ability of the nomogram is

Hazard ratios (HRs) calculated from multi‐Cox regression analysis suggested that the 6‐DNA methylation signature was an independent predictor for TPC patients' RFS (*P* < .001, HR 2.13, 95% CI 1.52‐2.99) (Table 2). Nomogram (Figure 7) that combined both the 6‐DNA methylation signature and other conventional clinical pathological factors yielded a significant *P* value in multivariate cox model to predict RFS. The importance of each factor was displayed in Figure 8A. The evaluative indicator such as C‐index (0.796, 95%CI: 0.704‐0.888), AUC (0.850, 0.783, 0.800) (Figure 8B), and calibration plot yielded a high predictive value simultaneously (Figure 8C‐E). The result suggested that the nomogram had high accuracy as a good model both in the training set and validation set as well as entire dataset, which strongly confirmed the reliability of the nomogram.

**Table 2:** Univariate Cox regression analysis and multivariate Cox regression analysis result based on methylation risk score as well as other clinical factors

Characteristics	Univariate analysis				Multivariate analysis			
	HR	HR.95L	HR.95H	*P* value	HR	HR.95L	HR.95H	*P* value
Score	2.1360795	1.522979101	2.995993595	1.10E‐05	1.798933288	1.099079897	2.944427411	.019503541
Sex	1.478175756	0.6586867	3.317212213	.343326328	1.100244516	0.46236439	2.618147118	.829003652
Histological type	1.102615623	0.414814152	2.930857599	.844731921	0.88338684	0.269481895	2.895824631	.837814763
T	1.955591733	0.897745673	4.259935907	.091331785	0.768819721	0.282591758	2.091652523	.606669818
N	1.576230647	0.72382004	3.43248724	.251804892	1.111798812	0.430884087	2.868745071	.826546152
M	13.18960301	3.08791975	56.33748338	.000497731	11.81173954	1.319254183	105.7545944	.027265308
Age	1.03059987	1.006178371	1.055614116	.013764397	1.021223872	0.993086952	1.050157989	.140665817
Residual tumor	5.178822071	2.24018622	11.97230739	.00011989	3.33526888	1.17841795	9.439790443	.023253263

## 4 DISCUSSION

Employing DNA methylation as a hallmark has a few merits over other molecular hallmarks: (a) DNA methylation marker is relative stable both in vivo and ex vivo.^18^ (b) A smaller amount of tissues are need to achieve sufficient DNA for the analysis of methylation.^19^ (c) Relative accuracy of DNA methylation due to quantitative assay as DNA methylation measurements can be compared with absolute reference points.^20^ Numerous evidence has revealed that DNA methylation signatures can predict the clinical prognosis of various types of cancer. For instance, one study reported that DNA methylation mediated the silencing of microRNA‐874 and can serve as a promising diagnostic and prognostic marker in breast cancer.^21^ Another recent study revealed that DNA methylation was an independent prognostic marker of survival in adrenocortical cancer.^22^ DNA methylation of the PITX2 gene promoter region serves as a strong independent prognostic marker of biochemical recurrence in patients with prostate cancer who had received radical prostatectomy.^23^ However, these investigations were limited by small sample size and lack of utilization of the biomarker as an independent prognostic signature. Several studies indicated that the combination of different DNA methylation as biomarker obtained better performance than individual DNA methylation.^19^, ^24^, ^25^ In this study, a 6‐DNA methylation signature showed good performance to predict RFS in patients with TPC. The 6‐DNA methylation marker also performed well in differentiating low‐ and high‐risk cohorts, suggesting that it was an independent predictor for TPC patients' RFS when adjusted by age, FIGO stages, histologic grade. In addition, the predictive performance for the combination of these 6‐DNA methylation sites was better than that for the six individual methylation sites in both training and validation datasets. Kaplan‐Meier analyses also implied that, compared to the six individual methylation sites, a combination of methylation sites had a better predictive value for TPC patients' RFS.

The screened six methylation sites were projected into four genes: DIO3, MIR1247, MCF2L, CCDC80. Researchers have reported that the above four genes were important in cancer development. The CL2/DRO1/CCDC80 served as tumor suppressor genes in thyroid carcinogenesis ^26^ The rho‐specific guanine nucleotide exchange factor DBS (MCF2L) can regulate breast cancer cell migration.^27^ The regulation of MIR941 and MIR1247 was associated with gastric cancer cell growth and migration.^28^ Those result revealed that the genes associated with these four sites played a key role in cancer progression.

The strengths of our studies were that we used LASSO method to filter variables between univariate and multivariate cox analysis, which solved the multicollinearity problem and makes our results more reliable. Besides, few previous studies have combined methylation signature with clinical indicators to predict RFS for TC yet. We combined methylation bioinformatics analysis with clinical indicators to develop a nomogram for predicting TPC patient's RFS, offering novel method for clinical prediction. In addition, Our nomogram was employed to predict the prognosis of TPC patients in a quantitative method, in other words, the nomogram can predict specific survival percentage of TPC patients, which may improve prognostic prediction for TPC patients.

Apart from the inspiring findings, the present study also has several limitations. First, we identified a 6‐DNA methylation signature only through the TCGA database due to the incomplete clinical data (RFS information) of thyroid cancer samples from GEO database or ArrayExpress database or ICGC database. Second, quite a long time is required for applying it clinically, due to high methylation testing charge. Third, our nomogram was constructed on the basis of retrospective data from TCGA database, which may generate hazard of selection bias.

## 5 CONCLUSION

Considering crucial connections with TPC patients' RFS, the 6‐DNA methylation may be a potential therapeutic target for TPC. Furthermore, a comparison of the 6‐DNA methylation signature with other known prognostic biomarkers showed that it had strikingly better performance in predicting TPC patients' RFS. Lastly, we constructed a nomogram that combined both the 6‐DNA methylation signature and the conventional clinicopathological factors to predict 1‐, 3‐, and 5‐year RFS. The result also contributes to the development of effective molecular markers for clinical practice.

## CONFLICT OF INTEREST

The authors declare that they have no competing interests.

## AUTHORS CONTRIBUTIONS

HC and XM designed, extracted, analyzed, and interpreted the data from GEO and TCGA databases. MY and MW wrote the manuscript. Lei Li and Kaixiong Tao made substantial contributions to the conception of the work and substantively revised it. All authors have read and approved the final manuscript.

## Supporting information

Fig S1

Fig S2

Fig S3

Fig S4

Fig S5

Fig S6

Fig S7

Fig S8

Fig S9

Table S1

Table S2

Supplementary Material

## Data Availability

All data generated or analyzed during the present study are included in this published article or are available from the corresponding author on reasonable request.
