# Supplementary figures and images for: A methylomics‐associated nomogram predicts recurrence‐free survival of thyroid papillary carcinoma
Source: Cancer Med. 2020 Aug 11;9(19):7183–93. doi: 10.1002/cam4.3388 (PMC7541134; doi:10.1002/cam4.3388)

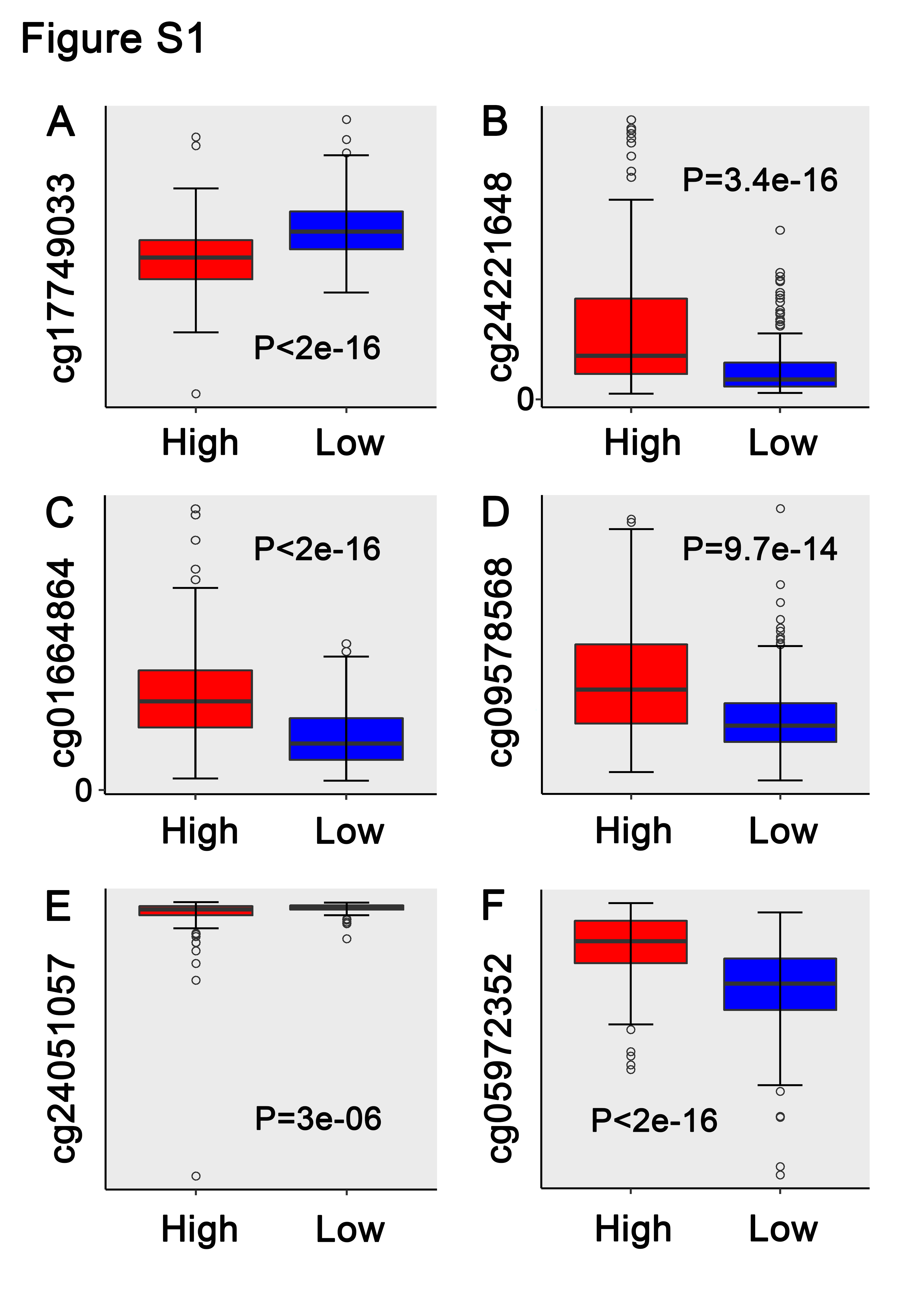

Supplement: Supplementary file 1 — Fig S1 [file CAM4-9-7183-s001.tif]

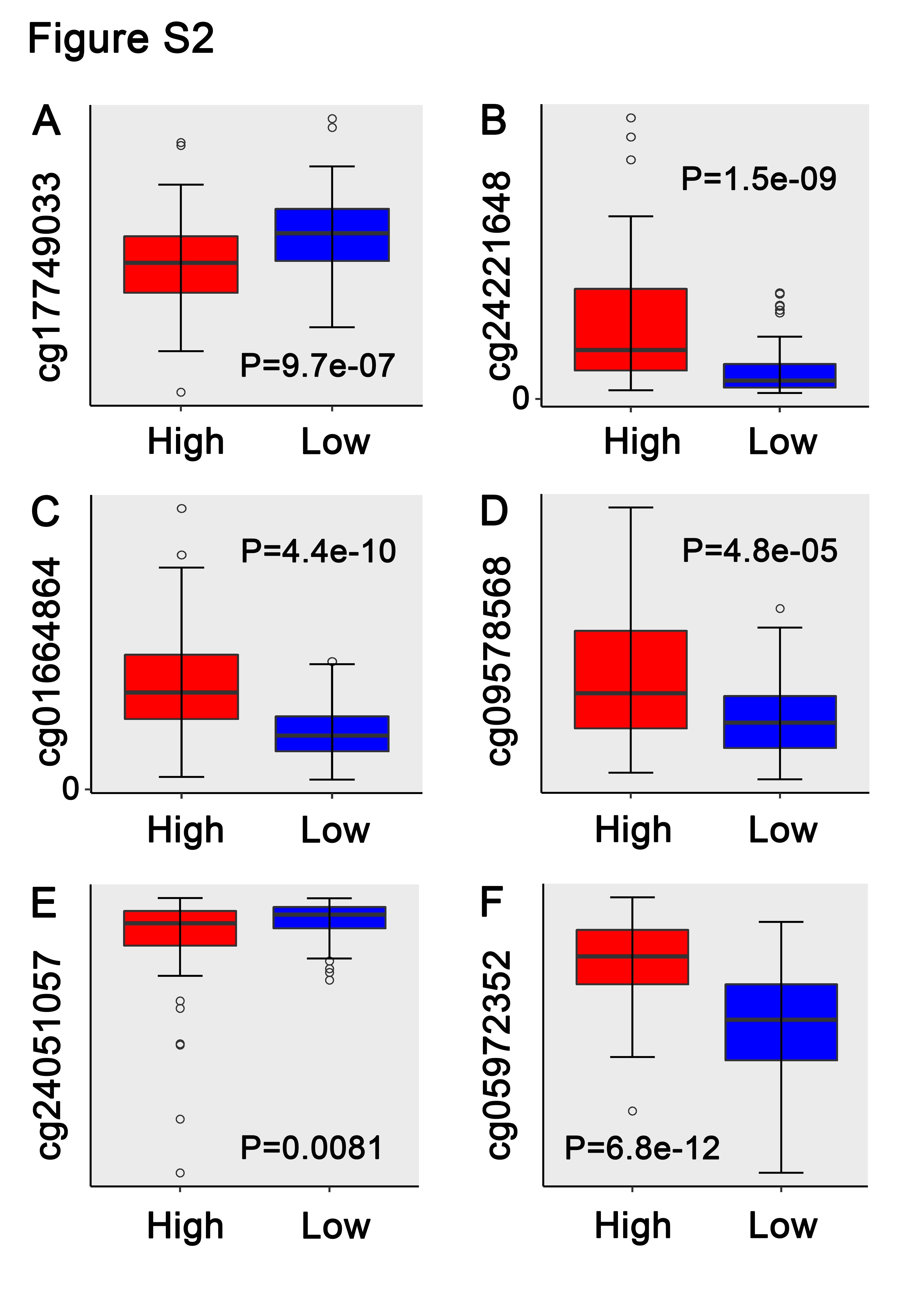

Supplement: Supplementary file 2 — Fig S2 [file CAM4-9-7183-s002.tif]

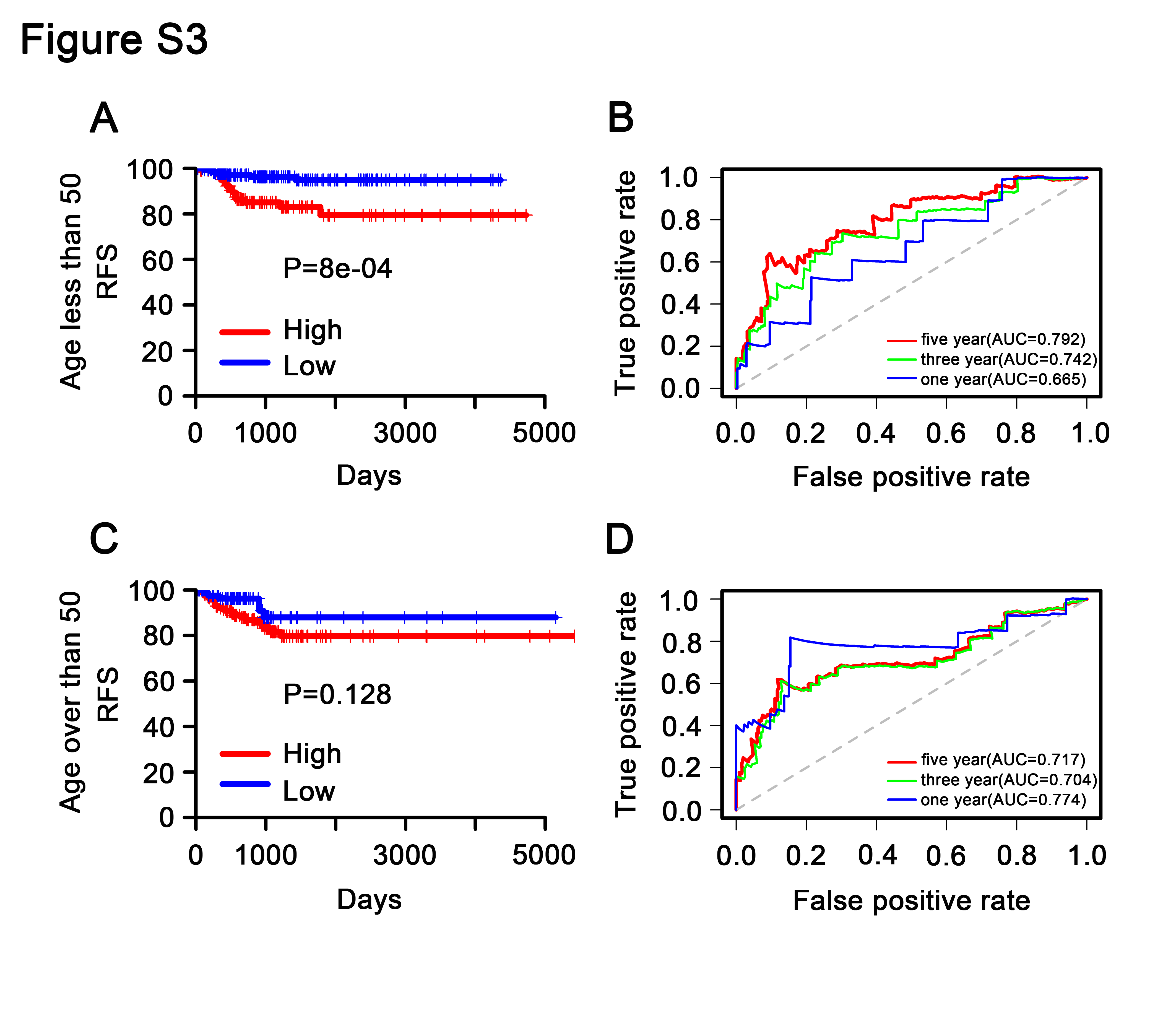

Supplement: Supplementary file 3 — Fig S3 [file CAM4-9-7183-s003.tif]

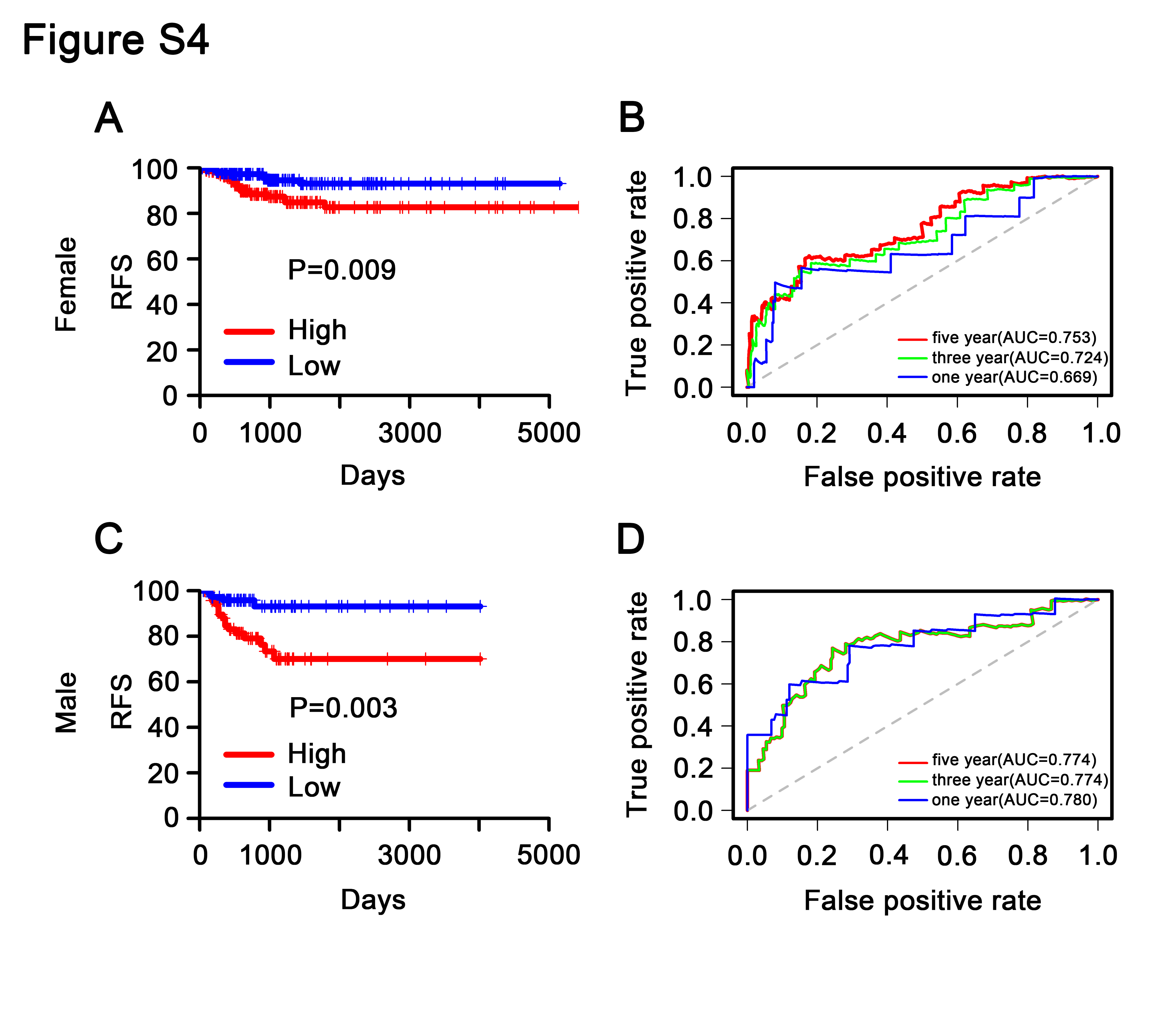

Supplement: Supplementary file 4 — Fig S4 [file CAM4-9-7183-s004.tif]

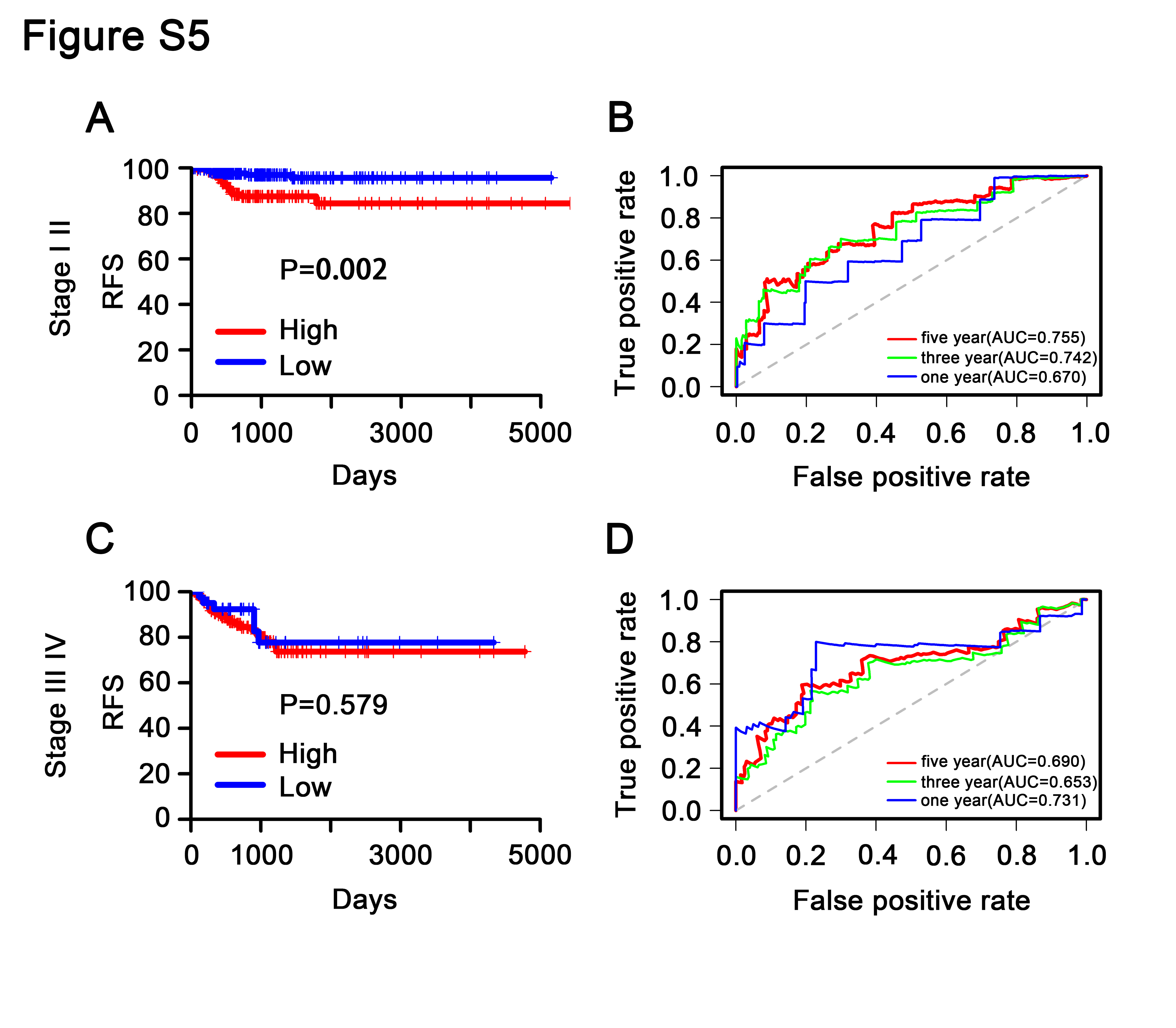

Supplement: Supplementary file 5 — Fig S5 [file CAM4-9-7183-s005.tif]

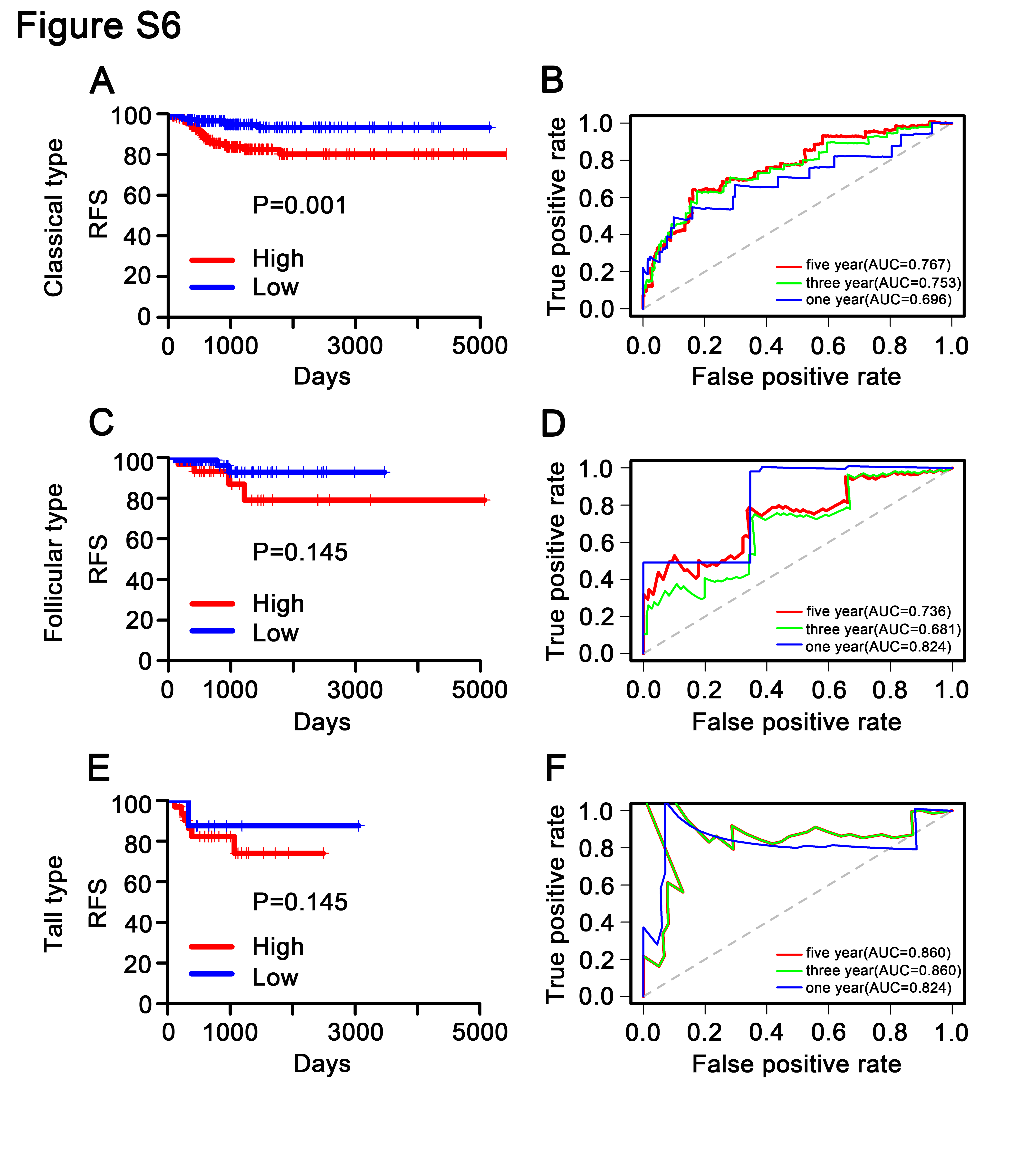

Supplement: Supplementary file 6 — Fig S6 [file CAM4-9-7183-s006.tif]

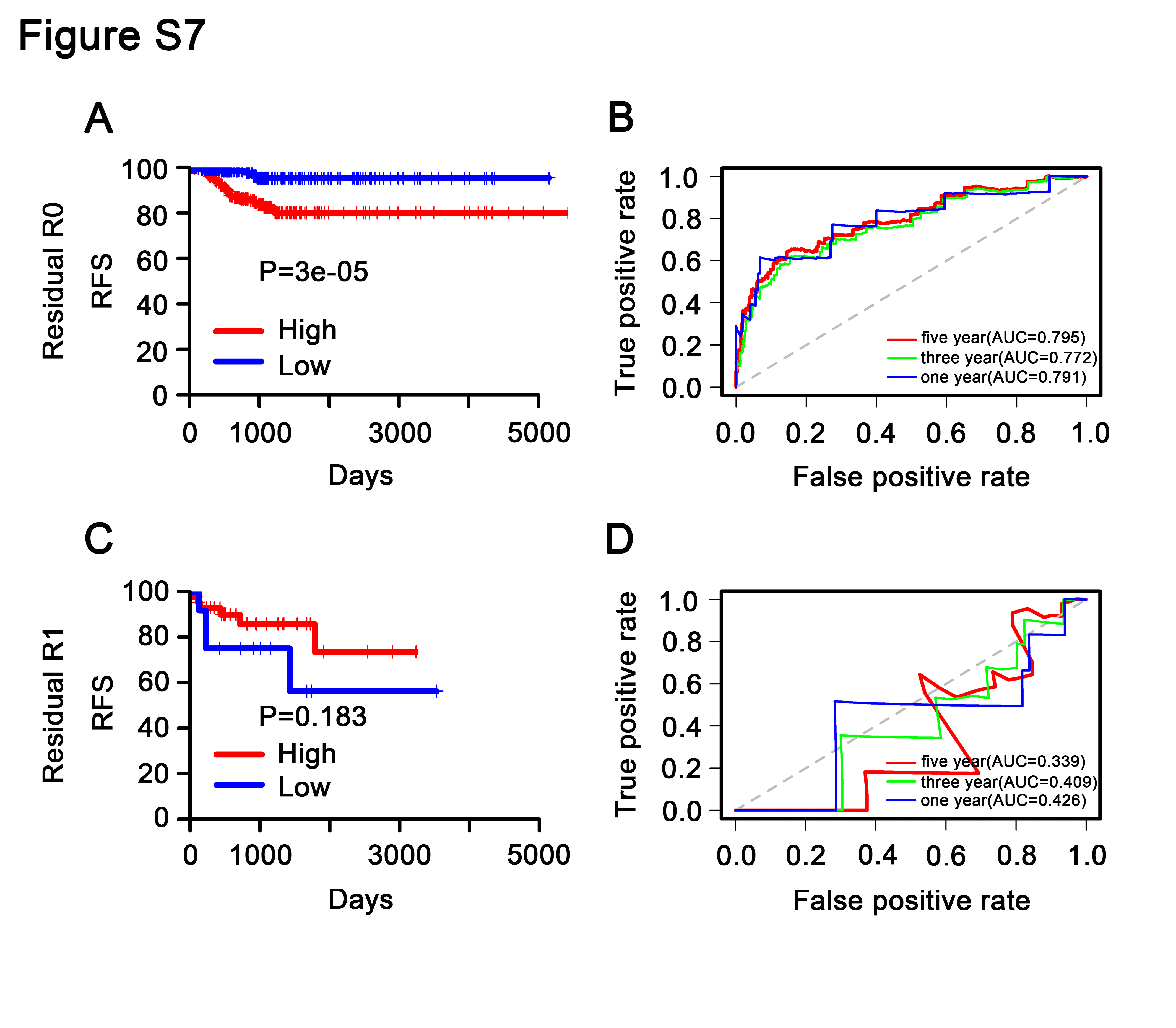

Supplement: Supplementary file 7 — Fig S7 [file CAM4-9-7183-s007.tif]

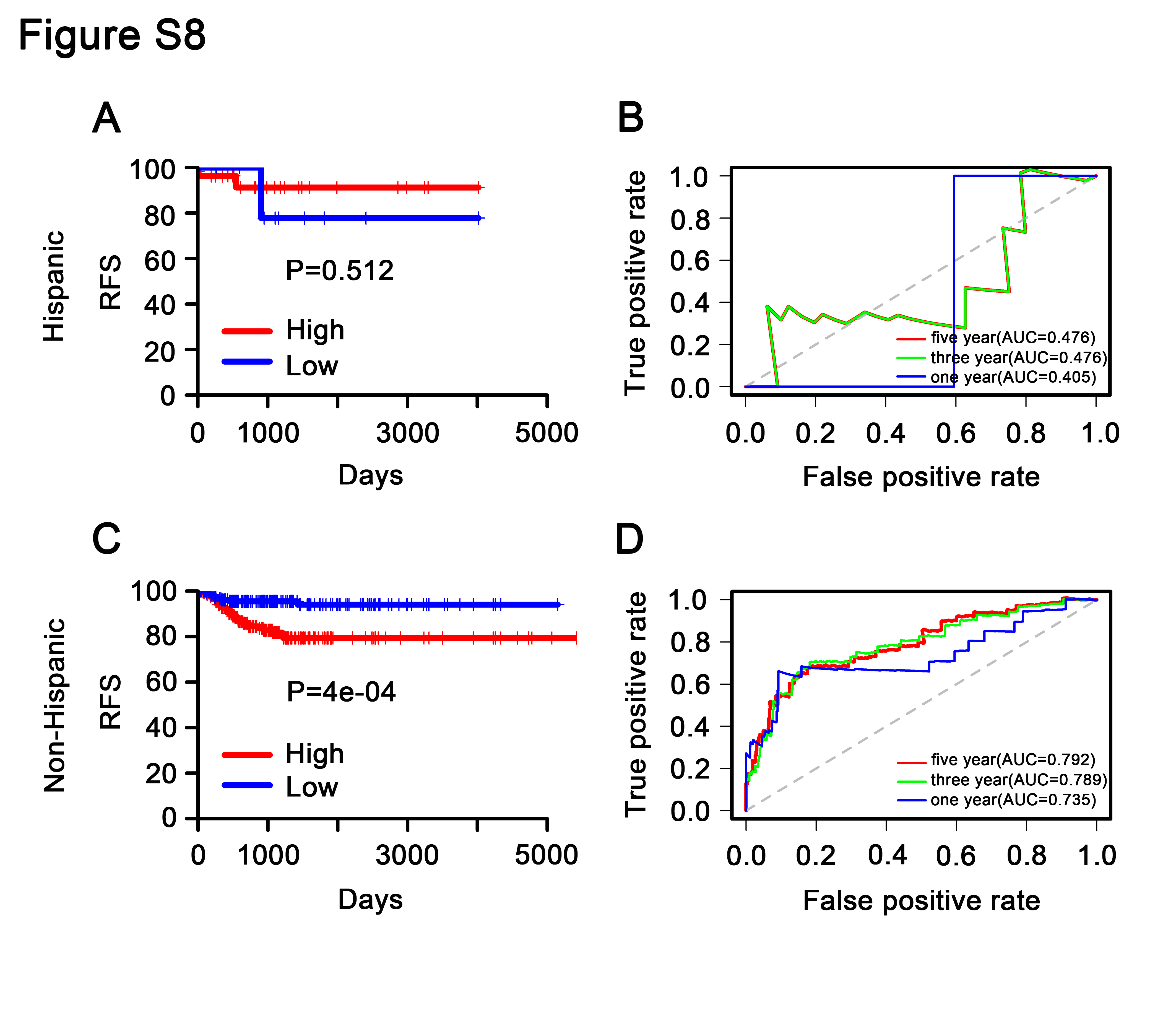

Supplement: Supplementary file 8 — Fig S8 [file CAM4-9-7183-s008.tif]

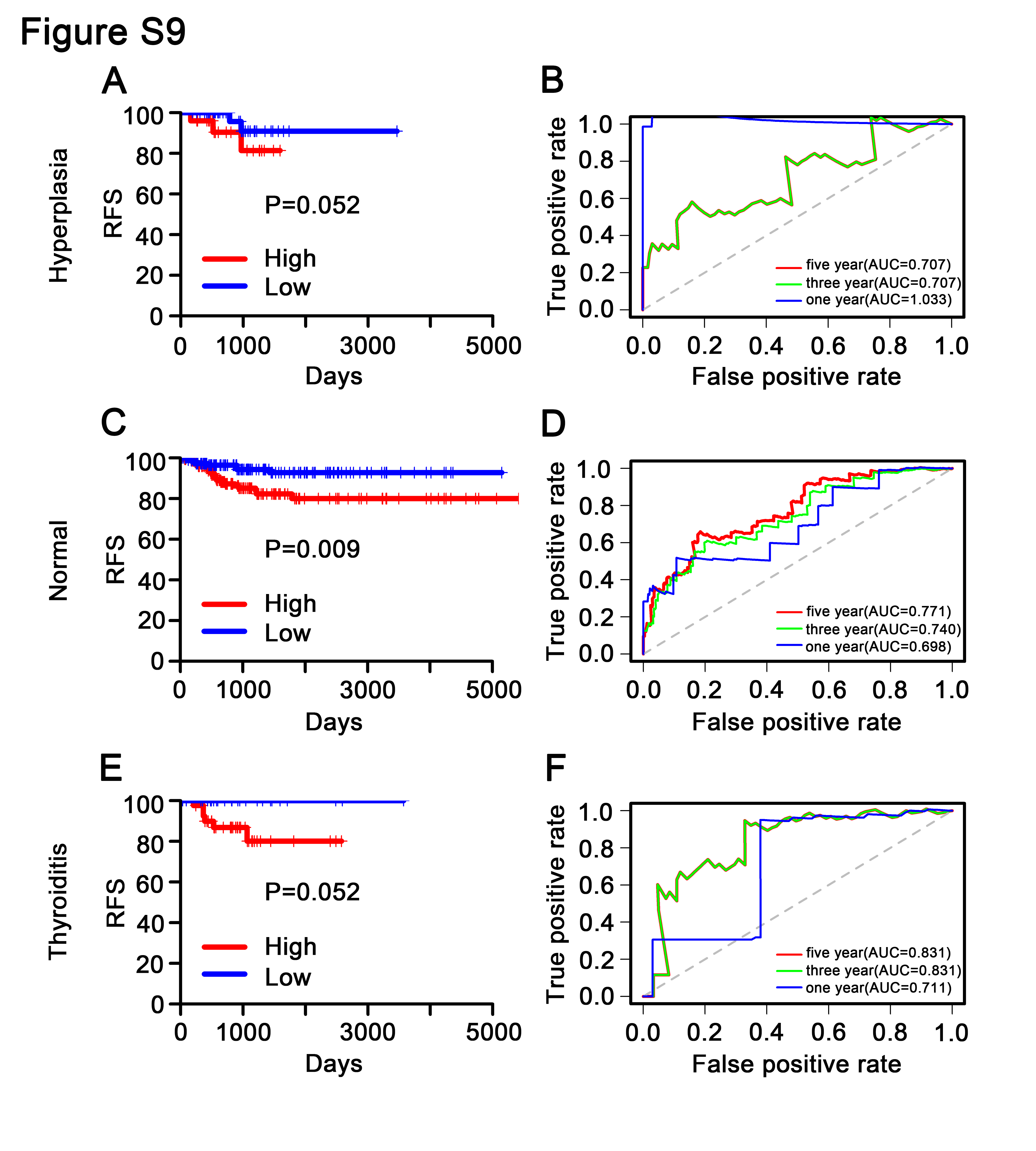

Supplement: Supplementary file 9 — Fig S9 [file CAM4-9-7183-s009.tif]
