# Supplementary material for: A methylomics‐associated nomogram predicts recurrence‐free survival of thyroid papillary carcinoma
Source: Cancer Med. 2020 Aug 11;9(19):7183–93. doi: 10.1002/cam4.3388 (PMC7541134; doi:10.1002/cam4.3388)
Supplement: Supplementary file 12 — Supplementary Material [file CAM4-9-7183-s012.doc]

**Supplementary Figure and Table Legends**

**Figure S1. Boxplots of methylation β values against risk group in the training dataset.**

**Figure S2. Boxplots of methylation β values against risk group in the testing dataset.**

**Figure S3. Kaplan-Meier and ROC analysis of TPC patients in sub-groups based on sex, respectively. A), B)** Female sub-group. **C), D)** Male sub-group.

**Figure S4. Kaplan-Meier and ROC analysis of TPC patients in sub-groups based on age, respectively. A), B) Age** ≤50 years sub-group. **C), D)** Age ≥ 50 years sub-group.

**Figure S5. Kaplan-Meier and ROC analysis of TPC patients in sub-groups via stage, respectively**. **A), B)** Early stage sub-group. **C), D)** Advanced stage sub-group.

**Figure S6. Kaplan-Meier and ROC analysis of** **TPC patients in sub-groups via histologic type, respectively.** **A), B)** Classical sub-group. **C), D)** Follicular sub-group. **E), F)** Tall sub-group.

**Figure S7. Kaplan-Meier and ROC analysis of TPC patients in sub-groups based on residual status, respectively.** **A), B)** R0 sub-group. **C), D)** R1/R2 sub-group.

**Figure S8. Kaplan-Meier and ROC analysis of TPC patients in sub-groups based on medical history, respectively.** **A), B)** Hyperplasia sub-group. **C), D)** Normal sub-group.

**Figure S9.** **Kaplan-Meier and ROC analysis of TPC patients in sub-groups based on ethnicity, respectively. A), B)** Hispanic sub-group. **C), D)** None- hispanic sub-group.

**Table S1. Hazard ratios and 95% CIs as well as P values of 1000 methylation sites based on univariate Cox regression analysis.**

**Table S2. 6 DNA methylation signature-correlated biological pathways.** ‘Corr’ stood for the Pearson correlations between the methylation risk score and GSVA score. ‘P value’ stood for significance of Pearson correlations.
